# Cross-sector perspectives on the role of a UK national cultural asset in social prescribing: a qualitative study

**DOI:** 10.1186/s12875-025-03156-7

**Published:** 2026-01-03

**Authors:** Emily Davis

**Affiliations:** 1 Department of Sport and Exercise Sciences, Durham University, Durham, UK; 2 Directorate of Research and Engagement, Royal Conservatoire of Scotland, Glasgow, UK

**Keywords:** Social prescribing, Arts on prescription, Cultural assets, Dance for health, Arts in health, Neurological conditions, Cross-sector collaboration

## Abstract

**Background:**

Recognition of the role of the culture sector in promoting health and well-being is increasing across research, practice, and policy. Despite this growing prominence, there remains more limited understanding of how cultural assets can be effectively coordinated with health services, or how these cultural provisions are perceived by professionals working across the health and third sectors. This study aimed to explore cross-sector professionals’ perspectives on the efforts of Scottish Ballet, a UK national cultural asset, to connect its dance provisions for neurological conditions into health and social care pathways through social prescribing.

**Methods:**

An exploratory qualitative study was conducted using semi-structured, online interviews with professionals across the health, cultural, and third sectors. Participants were purposively sampled, focused on three health boards: NHS Greater Glasgow and Clyde, NHS Tayside, and NHS Orkney. Interviews were transcribed verbatim and analysed using reflexive thematic analysis.

**Results:**

Fourteen professionals were interviewed. The analysis identified two themes reflecting the dual role of Scottish Ballet in relation to social prescribing: (1) as a credible cultural service *provider* of dance for people with neurological conditions, and (2) as a cross-sector *promoter* of the arts in health practice and policy. Sub-themes show opportunities for connecting dance provisions with health services by enhancing professionals’ delivery of person-centred care and expanding access to dance activity for certain patients with reduced mobility, alongside challenges with limited professional-level capacity, inconsistent service-level coordination, and lacking sustainable, system-level support.

**Conclusions:**

Cultural assets such as Scottish Ballet can offer meaningful contributions to social prescribing, both as direct service providers and wider strategic advocates. Yet, sustained collaboration, shared understanding, and long-term investment across sectors remain important to more comprehensively and sustainably embedding the arts and culture in prescribing pathways.

**Supplementary Information:**

The online version contains supplementary material available at 10.1186/s12875-025-03156-7.

## Background

Neurological conditions are now recognised as the leading cause of illness and disability globally [1]. These conditions are characterised by nervous system damage, but are diverse in disease aetiology, symptoms, and progression [2]. Certain chronic neurological conditions, such as dementia, Parkinson’s disease (Parkinson’s), and multiple sclerosis (MS), are degenerative in nature, resulting in the progressive loss of function over time [1]. Given most progressive neurological conditions lack a cure, they require long-term, comprehensive management to not only address physical impairments, but also the emotional and social consequences of living with a degenerative, and oftentimes, disabling illness [3, 4]. In the UK, current healthcare guidance recommends various person-centred pharmacological and non-pharmacological interventions to support the long-term management of long-term neurological conditions [5].

Non-clinical interventions such as physical activity programmes, community supports, and creative activities are increasingly being recommended by health professionals to support symptom management and health promotion among people with chronic neurological conditions [5–8]. Social prescribing has emerged as a key pathway for connecting these types of non-clinical interventions with health services, whereby health professionals ‘connect patients to community-based activities, groups, and services to improve their health and well-being by addressing social, emotional, and practical needs’ [9]. While social prescribing has gained traction internationally, the UK has arguably established one of the most formalised approaches, with the National Health Service (NHS) England Long Term Plan [10] committing to integrating social prescribing services and link workers across primary care practices.

Although NHS England set out this more standardised primary care-based approach, in practice, social prescribing varies across professional settings and regional contexts [11–13]. For example, in certain areas of England and Scotland, four different social prescribing models were identified, ranging from English primary care networks directly employing link workers to Scottish Health and Social Care Partnerships outsourcing their employment to the voluntary sector [12]. This diversity of models entails regional variation in social prescribing practice, including differences in self-referral options and the generic or specialist nature of provisions [12]. This variation reflects localised interpretations of social prescribing, with some arguing for further consideration of such localised approaches, viewing social prescribing as a diverse, adaptable ‘idea’ or ‘pathway rather than a coherent entity’ [13]. Aligning with this broader conceptualisation of social prescribing, interest is growing in exploring beyond core NHS, primary care-based models to consider the potential of non-NHS, community-based pathways, including the role of voluntary, community, and culture sectors [13].

The culture sector has played a key role as a service provider and partner in social prescribing, with ‘arts on prescription’ (also known as arts prescribing and arts on referral) emerging as a subset or complement to the wider social prescribing framework [14, 15]. Similar to wider social prescribing processes, arts on prescription connects patients to bespoke arts activities in community, third-sector, or health settings to improve their health and well-being [15–17]. Oftentimes, such initiatives can involve cross-sectoral partnerships between healthcare providers and cultural institutions, such as museums [18]. Certain arts provisions are targeted to specific health conditions, with dance for Parkinson’s and music for dementia being particularly well-established and well-evidenced in the context of arts and health [19, 20].

While a growing evidence base suggests the benefits of arts engagement for patient health experiences and outcomes [14, 17], arts on prescription effectiveness and implementation extend beyond patient participation, relying on professionals to adopt, prescribe, and integrate provisions within health and social care pathways [21]. Despite their central role, there has been limited attention towards understanding health professionals’ perspectives on coordinating the arts with health services via arts on prescription [18, 22]. Moreover, there has been limited consideration of professional perspectives across the culture, health, and third sectors regarding the changing roles, responsibilities, and implications for arts organisations as they transition from independent cultural providers to integrated partners in social prescribing pathways [18, 22, 23].

Especially as the arts continue to further connect with healthcare services via social and arts prescribing, there is a growing need to understand the perspectives of health, cultural, and third-sector stakeholders, including ‘the structures and the gaps in connecting all stakeholders’ [23]. Previous research has explored health professionals’ views on social prescribing directed by the health sector via core NHS primary care-based pathways [24, 25]; however, there remains more limited understanding of the potential of arts-based prescribing driven by the cultural sector and operating across both NHS and non-NHS, community-based routes. The current study aimed to explore cross-sector professionals’ experiences, perceptions, and understandings of Scottish Ballet’s developing dance-based prescribing model, providing insight into the barriers, enablers, and role of a national cultural asset connecting their dance health programmes for neurological conditions into mainstream health and social care pathways in Scotland.

## Methods

### Study context

Scottish Ballet, Scotland’s National Dance Company, launched as a National Centre for Dance Health in 2023, building on over a decade of experience in providing dance-based health and well-being programmes across generations [26]. Scottish Ballet’s health initiative, SB Health, designed and delivers three flagship neurological programmes in their Glasgow, UK headquarters: Dance for Parkinson’s Scotland, Time to Dance^®^ (dementia-friendly programme), and SB Elevate^®^ for people with MS. Scottish Ballet also operates nationally via virtual and hybrid engagement, alongside supporting in-person neurological offerings in Tayside, UK and Orkney, UK. As part of long-term efforts to more formally coordinate their programming with health and social care in Scotland, Scottish Ballet, in collaboration with the national third-sector intermediary, the Health and Social Care Alliance Scotland (the ALLIANCE) [27], led a three-year strategic project aimed at expanding access to their existing neurological provisions via developing ‘dance on prescription’ pathways.

**Fig. 1:**
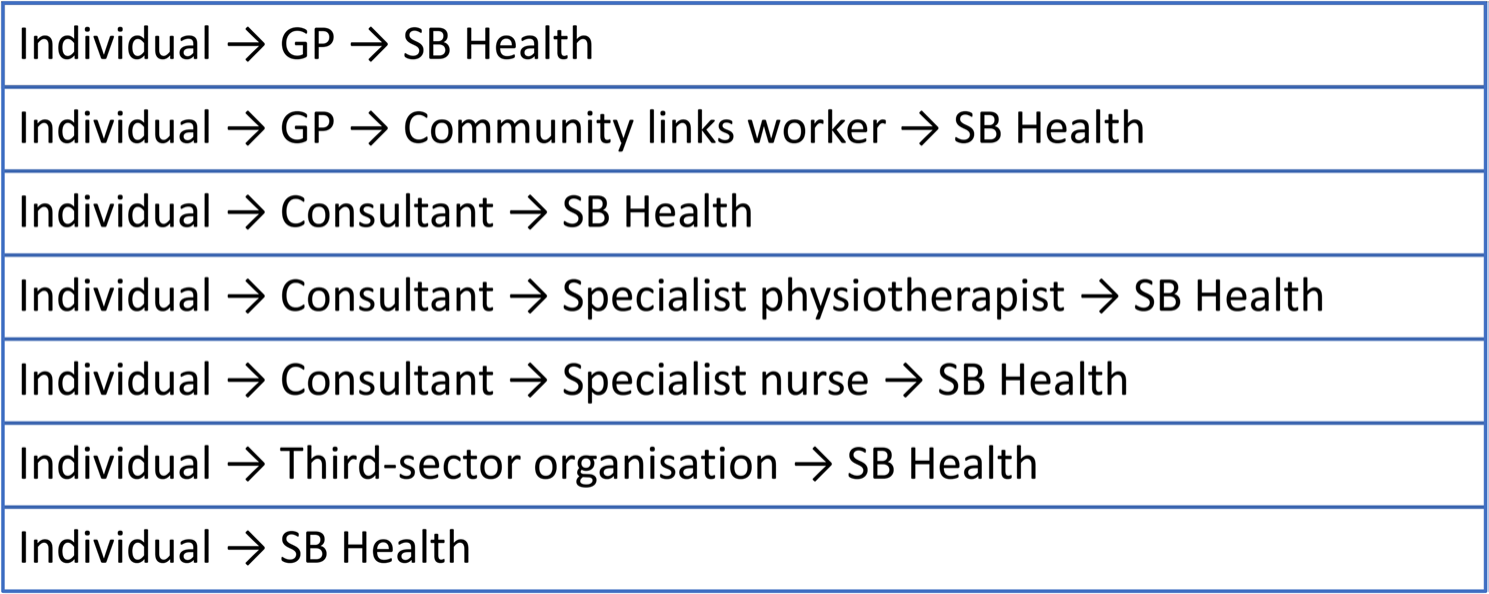
Pathways to SB Health

A key aspect of this collaboration and pathway development was Scottish Ballet’s employment of a Health Partnerships Manager, also supported by the ALLIANCE, with responsibility for building connections between Scottish Ballet and health and social care. Scottish Ballet and their Health Partnerships Manager focused their resources on the three NHS health board areas with in-person SB Health neurological provisions: NHS Greater Glasgow and Clyde (GGC), NHS Tayside, and NHS Orkney, to better understand localised social prescribing priorities, needs, and pathways. In each region, self-motivated self-referral remained an option for patients, but exploring further cross-sector pathways between NHS primary and secondary care, the third sector, and Scottish Ballet was a priority for the Health Partnerships Manager and the project overall. Given the project’s context-specific, exploratory, and relational nature, a range of delivery pathways were developed (Fig. 1).

These pathways’ design and delivery varied across the different urban, semi-rural, and remote regional contexts and health settings, from the largest to the smallest health board in Scotland [28, 29]. In NHS GGC, Scotland’s largest health board, signposting of SB Health’s provisions was encouraged top-down across the ALLIANCE’s well-established Community Links Worker Programme, embedded across 80 Glasgow primary care practices [30]. In NHS Tayside, a mid-sized Scottish health board, Scottish Ballet devised a more structured active signposting pathway via secondary care. This included the collaborative development of a digital referral form and paper information sheets used by professionals, from consultants to administrators, to signpost patients to neurological offerings. In Scotland’s smallest health board, Orkney, Scottish Ballet secured funding from the local NHS endowment or charitable fund to sponsor dance session delivery and promote active signposting, primarily among primary care professionals who advised on appropriate pathways. Across these regions, the Health Partnerships Manager also led awareness-raising sessions with different professional groups, including neurological consultants, general practitioner (GP) cluster teams, and allied health professionals.

### Study design

An exploratory qualitative interview study design was employed, using one-to-one, semi-structured interviews to examine health, culture, and third-sector professionals’ perspectives on Scottish Ballet’s developing dance-based prescribing model. This study formed part of a focused external evaluation around professional perspectives on Scottish Ballet’s role and model by the author [ED], distinct from Scottish Ballet’s broader internal project evaluation. The semi-structured interview approach allowed for in-depth exploration of individual perspectives on key topics while maintaining flexibility to probe emerging lines of inquiry [31]. ED conducted interviews online to accommodate participants’ professional schedules and different geographic locations. The study followed the Consolidated Criteria for Reporting Qualitative Research (COREQ) 32-item checklist [32] and was conducted in accordance with the Declaration of Helsinki. Ethical approval for the study was granted by the Royal Conservatoire of Scotland Ethics Committee in July 2024 (Reference number: EC2378).

### Study sample

Participants were recruited through purposive sampling via Scottish Ballet’s personal networks, including health professionals (clinical and non-clinical), third-sector professionals, and key project stakeholders from Scottish Ballet and the ALLIANCE, with direct involvement with or close understanding of Scottish Ballet’s developing dance on prescription pathways. This purposeful approach allowed for a sample with in-depth expertise around Scottish Ballet’s developing social prescribing approach, while also maximum variation across professional roles and contexts [33]. Scottish Ballet facilitated initial email contact, providing potential participants with study information and requesting permission to share their details with ED. Subsequently, ED emailed formal invitations, participant information sheets, and consent forms to participants (*n* = 20). Recruitment occurred over 12 weeks, including bi-weekly follow-up emails to encourage participation, with several not responding (*n* = 6). Fourteen participants participated in the study, all providing voluntary written informed consent before participation.

### Data collection

Interviews were conducted by ED between November 2024 and January 2025 using Microsoft Teams, lasting between 35 and 70 min. ED is a female researcher with a PhD in dance health, trained and experienced in qualitative interviewing. She had no prior relationship to twelve participants and a working relationship with the other two participants, two Scottish Ballet staff members. The semi-structured interview guide employed focused on understanding professionals’ experiences with developing and delivering dance on prescription, their perceptions of Scottish Ballet’s coordination with healthcare, and broader reflections on barriers and enablers in Scotland’s social prescribing landscape (Supplementary Table 1). All interviews were audio-recorded following participant permissions and transcribed verbatim with assistance from NoScribe [34], a remote AI-powered transcription software.

### Data analysis

Interview transcripts were imported into NVivo software (QSR International, Melbourne, Australia, Version 14) for analysis. Numbering and roles replaced participant names. Reflexive thematic analysis was utilised, following Braun and Clarke’s six-phased approach [35]: (1) data familiarisation, (2) coding, (3) generating initial themes, (4) reviewing potential themes, (5) defining and renaming themes, and (6) report writing. Coding was conducted inductively, deriving themes directly from the data while aiming to maintain descriptive and interpretive validity [36]. Initial codes were aggregated around shared patterns of meaning, yielding initial themes, which were iteratively developed and refined in a process guided by the research aim. Rigour in analysis and interpretation was maintained through ongoing reflexivity, including reflective memo-writing, critical consideration of positionality, and continuous questioning of the validity and reliability of codes, themes, and interpretations [35]. Rigour in the reporting was enhanced by the inclusion of a transparent audit trail of codes, themes, sub-themes, and supporting quotations in the results [32, 35].

## Results

Fourteen participants took part in the study. Most participants were health sector professionals (*n* = 7), with representation from the third sector (e.g., the ALLIANCE; *n* = 5) and cultural sector (i.e., Scottish Ballet; *n* = 2). The sample was balanced between those in more strategic or executive roles (*n* = 7), both in clinical and community settings, and those in more frontline, patient-facing positions (*n* = 7), such as health professionals (e.g., consultants) and allied health professionals (e.g., physiotherapists). A few professionals (*n* = 4) also held voluntary strategic roles with SB Health (e.g., internal advisory committee members), alongside their active positions in the health sector and the developing dance on prescription model. Participants also reflected some geographic diversity, with more representation from urban, i.e., Glasgow (*n* = 8), than semi-rural and remote regions, i.e., Tayside and Orkney (*n* = 4). Only certain participant characteristics are detailed to protect anonymity (Table 1).

**Table 1:** Number of participants by role

Participant Role	Number of Participants
Third Sector Executives (TSE)	3
Scottish Ballet Staff (SBS)	2
Health Executives (HE)	2
Health Professionals (HP)	3
Community Links Professionals (CLP)	2
Allied Health Professionals (AHP)	2

Across participants, Scottish Ballet was perceived as functioning at two levels in relation to social prescribing, at an operational-level, delivering dance health provisions for neurological conditions while developing pathways towards ‘dance on prescription,’ and at a strategic level, promoting wider cross-sector coordination between culture and health in Scotland, including via social prescribing. Two themes emerged in line with these dual roles: Scottish Ballet as a service ‘provider’ and Scottish Ballet as a strategic ‘promoter,’ with sub-themes further characterising professionals’ understandings and experiences of these roles (Table 2)

**Table 2:** Summary of findings

Theme	Sub-theme	Role Descriptor	Illustrative Codes
Scottish Ballet as a provider: implementing dance in health	Positioning dance provisions in healthcare	Clearly positioning dance provisions to health sector priorities encouraged leadership buy-in and institutional support for developing dance on prescription pathways, still influenced by the size, scope, and nature of each health service context and their social prescribing approach.	Building on established cross-sector partners; Health Partnerships Manager role; Fit-for-service priorities; Leadership/top-down support; Tailored signposting pathways; Diversity of signposting pathways; Healthcare capacity constraints
	Encouraging adoption among health professionals	Generally, dance provisions were positively perceived among health professionals, but practical adoption and prescribing of dance relied on close understanding and the utility of provisions, with strongest engagement among certain secondary care specialists with first-hand experience of offerings.	Perceived credibility of provisions; Addressing professional interests; Confidence in signposting; Key first-hand experiences; Relevance to professional practice; Convenience of signposting process; Levels of patient interest & uptake
	Widening patient access	Professional endorsement of dance provisions through prescribing processes enhanced uptake for some patients with reduced mobility; however, cultural perception, finance, and transport barriers still limited access for those in more remote and deprived areas.	Role of professional endorsement & encouragement; Cultural perception barriers; Financial & transport limitations; Hybrid access options (online/in-person); Key community-based access points; Culture sector capacity constraints
Scottish Ballet as a promoter: embedding dance in the health system	Advocating for arts and health	Scottish Ballet was viewed as holding an important leadership role in bridging the arts with health in Scotland, entailing wider responsibilities in maintaining their cultural positionality and amplifying broader cultural capacity.	Innovative leadership; Power of Scottish Ballet brand; Positive/proactive ethos of Scottish Ballet; HAS event as strategic platform; Piece of social prescribing puzzle; Tension in role responsibilities/ boundaries
	Leveraging strategic relationships	Key strategic relationships, roles, and structures helped Scottish Ballet build credibility and networks within the medical system, with some urging for a further systems-level orientation beyond social prescribing.	Reliance on third-sector partners; Influence of internal structures; Individual professional advocacy; Limited ‘pocket’ of partners; Limits of individual-level approach; Limits of social prescribing approach
	Maintaining cross-sector progress	Challenges remained in connecting Scottish Ballet’s provisions with health services at a systems-level owing to limitations in cross-sector evidence standards, funding availability, and regulatory structures.	Evidence & evaluation challenges; Mismatched governance standards; Siloed health system; Funding insecurity; Limiting ‘nice to have’ perception; Cross-sector funding; Risks of arts-health coordination

### Scottish Ballet as a provider: implementing dance in health

#### Positioning dance provisions in healthcare

 From the inception of developing dance on prescription pathways, Scottish Ballet drew on the expertise and contacts of established partners in the health and third sectors to position their dance provisions in health settings, namely the ALLIANCE and members of their internal research and advisory committees. Specifically with ALLIANCE support, Scottish Ballet employed their Health Partnerships Manager, who most participants viewed as an “essential” [TSE] intermediary between SB Health’s provisions and healthcare providers.

*As a dance company*,* having a health professional embedded within our dance health team was a very new approach for us*,* and our Health Partnerships Manager comes with over two decades of experience of working in health and social care*,* so*,* for us*,* it was a really new opportunity to help us to navigate the system in lots of different ways and to broker those connections. *(Scottish Ballet Staff 1)

These key cross-sector partners, including the Health Partnerships Manager, helped Scottish Ballet align their approach to relevant health priorities of different professional groups and regional services, directly using terminology like “realistic medicine” [HE] and “social prescribing” [HP]. Several professionals working across sectors expressed that tailoring terminology when initially raising awareness among health professionals was “savvy” [TSE], helping translate the value of dance to health.

*Working through non-trained staff*,* trained staff*,* right through to Consultants and then to Chief Executives*, [the Health Partnerships Manager] *can speak in a way that will appeal to them and they will be able to understand everything that we do at dance health. *(Scottish Ballet Staff 2)

*They’re loud and clear on our priorities and it fits with our vision as an organisation. It fits in our corporate strategy and clinical strategy. *(Health Executive 1)

This strategic positioning facilitated buy-in from certain health professionals in more strategic or leadership positions in the three areas. Developing these shared priorities, objectives, and understandings between Scottish Ballet and regional NHS leadership, whether at the organisational level (e.g., executive leadership) or departmental level (e.g., clinical leadership), was explained by professionals in more strategic roles as a key determinant of the development and delivery of dance-based prescribing pathways in a given context.

*Does an organisation and a health board see this* [social prescribing] *as important or not? If you have leadership who really value it*,* then it’ll happen in my experience. *(Health Executive 1)

Still, across the three health boards, only secondary care within NHS Tayside established a more structured, active signposting pathway, including a bespoke digital referral form regularly used by specialist consultants, nurses, physiotherapists, and administrators. In Glasgow and Orkney, SB Health provisions were connected into pre-existing primary care-based social prescribing infrastructure, but signposting reportedly remained largely ad-hoc, inconsistent, and reliant on buy-in from individual NHS and non-NHS professionals, such as community links professionals. Consequently, while multiple NHS and non-NHS pathways were explored, the more tailored, specialised secondary care-based model seemed to result in stronger professional engagement than the more generic primary care-based approach.

*In Scotland*,* the most common social prescribing pathway or mechanism is within primary care*,* and it’s via the links worker route. While that is a really helpful pathway for us*,* it’s not the only pathway into our work and out of our work. *(Scottish Ballet Staff 1)

Especially in the “massive” [AHP] Glasgow context and the small Orkney service, health professionals described competing pressures as limiting their capacity to engage with non-traditional provisions like dance. In these contexts, pressurised conditions, such as increasing budget constraints, workload demands, and staff shortages, reportedly made dance on prescription difficult to implement, especially given the reliance on buy-in from individual professionals without service-level agreements.

*Maybe there is a way for further integrating into the health service. But I suppose*,* I know our resources are so limited*,* I don’t know what that would look like* […] *the service is massive*,* it can be quite challenging to know who to talk to. *(Allied Health Professional 1)

#### Encouraging adoption among health professionals

Overall, among those interviewed across various professional roles, sectors, and levels of proximity to SB Health, Scottish Ballet was viewed as offering meaningful dance provisions that support the health and well-being of individuals with neurological conditions. This generally positive perception was reportedly influenced by Scottish Ballet’s longstanding reputation in the culture sector and their developing credibility in the health sector for delivering high-quality, evidence-based dance health programmes.

*You’re not starting with a ‘Mickey Mouse’ organisation and just an idea. You’ve got a very concrete idea with meaningful outcomes. *(Health Professional 1)

For most professionals interviewed across sectors, the dance provisions also aligned with their professional values, providing what was perceived as a needed, person-centred, and health-promoting alternative to the prevailing “industrialised” [TSE], crisis-driven model of healthcare. For some health and third-sector professionals, the provisions also aligned with their personal interests, with several having a pre-existing engagement with Scottish Ballet or an appreciation for dance. Accordingly, those professionals most receptive to Scottish Ballet’s provisions were already open and bought into dance as a “new” [HP], non-medical approach to care.

*The NHS is supposed to be a health service. It’s actually a disease service. So everything we do is to promote health – and I’m sure dance is a very good example. *(Health Professional 3)

While most professionals shared positive perceptions, adoption by frontline professionals responsible for “doing the doing” [HE] of prescribing was affected by their understanding and confidence in Scottish Ballet’s provisions. Health professionals with first-hand experience, having attended live regional dance sessions, described sufficient understanding of the logistics, experience, and benefits to recommend the provisions to patients. However, second-hand awareness-raising efforts, such as information sessions led by the Health Partnerships Manager, were reportedly insufficient, with prescribing remaining “difficult to imagine” [CLP] in practice.

*After attending and experiencing it first-hand*,* I was able to*,* I suppose*,* promote it better and from there*,* I’ve obviously become more confident in the fact I can say to people – look*,* I have attended*,* I’ve seen it first hand*,* that people all abilities attended that group*,* and it’s accessible for all. So that has been helpful. *(Allied Health Professional 2)

The utility and perceived relevance of Scottish Ballet’s offerings to each professional’s role, practice area, and patient population, as well as the convenience of the signposting process, were also explained by health professionals as influencing adoption. Specifically, for secondary care neurology specialists, SB Health’s neurological offerings were considered more relevant than for primary care link workers serving a broader patient population.

*As a condition alone*,* I don’t tend to work with a lot of people with Parkinson’s*,* I don’t know if that* [Dance for Parkinson’s provision] *could be widened to any kind of condition? *(Community Links Professional 1)

Professionals’ dance-based prescribing was also influenced by patient responses and follow-up feedback. Frontline professionals who encountered limited enthusiasm or engagement from patients were reportedly less certain about the effectiveness of provisions. Conversely, positive feedback, specifically reports of improved physical activity, self-management, and social connection, was noted as reinforcing health professionals’ confidence in dance health, encouraging continued prescribing.

*The effect of being fit or being healthy is as good as some of the first-line drugs that we’ve used over the years. The magnitude of the benefit is really very large*,* and without it*,* people can be stuck in their house. It’s more than a luxury I think*,* it empowers patients to take control over their own self. *(Health Professional 1)

#### Widening patient access

 The reach of dance-based prescribing, and in turn, the dance provisions, was also shaped by the patient populations that health professionals served and signposted. For some patients, the professional-led signposting, such as via trusted primary or secondary care professionals, was reportedly seen as helping reduce access barriers compared to self-referral. Health professionals especially noted this for patients with advanced mobility limitations, who benefitted from additional professional encouragement and reassurance.

*It’s not just a dance class*,* it’s actually good for you and a GP* [laughing], *recommends this. If*,* you know*,* a health professional recommends*,* it just carries another little bit of weight in persuasion. *(Health Professional 3)

However, some professionals across sectors expressed concerns around an “inverse care law” [SBS], whereby access to quality care may ‘vary inversely with the need for it in the population served’ [37]. This was particularly noted by frontline professionals working with individuals in certain more remote and socioeconomically deprived areas, where Scottish Ballet, and ballet more broadly, was often perceived as “not for them” [CLP]. These cultural perceptions resulted in challenges in uptake, even with professional encouragement, and were reportedly compounded by additional physical, financial, and transportation access barriers.

*When I mentioned ballet*,* people were like*,* ballet? And to men*,* they were like no definitely not. So it’s just trying to cut through people’s perceptions of things.* (Community Links Professional 1)

To improve access equity, SB Health developed a hybrid delivery model, enabling prescribing to in-person and online offers. Certain health professionals noted this expanded access for some individuals, particularly those with advanced mobility limitations, but the virtual option was not necessarily viewed across sectors as an equitable substitute for in-person offers. Some health and third-sector professionals suggested further hyper-localised, in-person community access points and supports would be more effective in supporting equitable engagement.

*Fatigue and mobility issues are a big feature* […] *So that can limit people how accessible things are*,* they will not consider traveling far for a class. And it’s great that it’s online*,* but we can’t underestimate the importance of doing things in person and the benefits it brings. *(Allied Health Professional 2)

Yet, in widening in-person community access points, concerns were also raised about Scottish Ballet’s own capacity to expand provisions across the three regional areas while maintaining quality. Several professionals holding more strategic positions noted that scaling up to meet demand from prescribing pathways would require significant investment in staffing, training, and programme coordination to ensure consistent quality of delivery. Overall, professionals working across sectors and levels of influence expressed uncertainty around the supply, demand, and sustainability of scaling dance health provisions.

*I think Scottish Ballet is in a bit of a bind at the moment – in that*,* it is a centre of excellence*,* and so it wants to lead excellent classes that are led by excellent dancers and tutors. And I applaud that*,* obviously*,* but I’m not sure how sustainable it is. *(Third Sector Executive 1)

### Scottish Ballet as a promoter: embedding dance in the health system

#### Advocating for arts and health

 Scottish Ballet was also viewed as acting at a wider strategic level as a promoter for connecting arts and culture with health. Particularly, those interviewed in more executive positions perceived Scottish Ballet as a “visionary” [HP] and “proactive” [HE] leader in promoting more person-centred, arts-based approaches across the health system, taking “responsibility” [HP] for shifting the system rather than waiting for the system to change.

*You wouldn’t necessarily say it’s* [Scottish Ballet’s] *responsibility*,* but they have taken the responsibility of doing it. I think they are genuinely pretty bold and pretty visionary around it in a place where others are failing or don’t see it as their job to do. *(Health Professional 2)

A key reference to this leadership made by nearly all participants was the Healing Arts Scotland (HAS) 2024 event [38], where Scottish Ballet collaborated with the Jameel Arts and Health Lab (partnership of New York University, World Health Organization (WHO) Regional Office for Europe, Culturunners, and Community Jameel) to produce a national arts and health festival. During HAS 2024, Scottish Ballet brought together hundreds of local arts and health practitioners and organisations and hosted policy roundtables, providing a “platform” [TSE] for networking, showcasing Scotland’s diverse arts and health activity, and advancing national conversations around the role of the arts in health and social prescribing.

*Through Healing Arts Scotland*,* Scottish Ballet was showing how they’ve obviously got the certain qualities and skills to act as this host or even platform* […] *It enabled a lot of activities to take place and then it also brought people together to shape the conversation and involve other organisations and other players within Scotland. *(Third Sector Executive 1)

While Scottish Ballet’s leadership within the cultural sector was widely acknowledged, some professionals across sectors, especially those in strategic roles, felt there was an opportunity for Scottish Ballet to expand its focus on broader promotion across the health, culture, and third sectors rather than direct service delivery, building on the “momentum” [HE] from HAS 2024. Several expressed that this broader promotional focus could also address the aforementioned scaling concerns by supporting and amplifying other community-based cultural assets.

*It’s taking a higher*,* more strategic approach*,* rather than getting bogged down with how many classes to be run and who can come. *(Health Professional 3)

*How can they grow what’s already there in communities and give it a lift up*,* rather than feel they’ve got to kind of go create a specialist programme that only Scottish Ballet can deliver across the country? *(Third Sector Executive 1)

That said, certain professionals in more operational positions raised concerns about Scottish Ballet extending too far beyond their provider role in the culture sector. While professionals working across sectors recognised cultural institutions like Scottish Ballet as valuable partners in health promotion and social prescribing, some also expressed caution about the potential risks of arts organisations assuming too much health responsibility and overstepping their expertise and positionality in social prescribing pathways. These concerns were particularly noted in relation to established roles in the health sector, such as community links workers, suggesting the need for boundaries in responsibilities between arts and health professionals in both provision and promotion.

*Definitely culture and art play a big part in people’s health and wellbeing*,* and I think we should continue that kind of collaboration and bringing them together*,* because I think they can work hand in hand. But I think that delivering SB Health is very different to being a community links worker. *(Community Links Professional 2)

#### Leveraging strategic relationships

 Behind Scottish Ballet’s role as a more strategic actor was their own strategic relationships, particularly with the ALLIANCE, which provided support for their Health Partnerships Manager. This Health Partnerships Manager role was seen by project stakeholders as important not only for building the relational infrastructure to develop dance on prescription pathways, but also for developing strategic relationships to promote broader system-level change.

*Whether it’s about how you deliver services to people or whether it’s about how you change the system*,* it’s ultimately about the relationships that people can make. That’s the currency we have to try and create change. How well can we build relationships? So having the* [Health Partnerships Manager] *role was pretty essential. *(Third Sector Executive 2)

Scottish Ballet’s other strategic partners and structures, including its internal SB Health steering and research committees, were also viewed by professionals across sectors as playing a key role in promoting Scottish Ballet at a systems-level, often acting as a kind of “Inside Man” [HP]. These internal structures supported Scottish Ballet’s broader promotional efforts, for example, encouraging medical student placements, identifying funding opportunities, and “raising their profile” [HE] with influential stakeholders like policymakers. These strategic connections were noted as especially valuable in Scottish Ballet navigating and securing credibility within the more hierarchical medical “establishment” [HP].

*It’s helpful to have someone who’s part of the medical*,* I would call*,* establishment*,* opening doors for what is seen as kind of a bit quirky in terms of a Cultural*,* big C*,* approach to health creation. *(Health Professional 2)

More directly, these strategic relationships also created ripple effects within healthcare and third-sector networks. Health and third-sector professionals closely engaged with SB Health, particularly those with advisory roles, became active advocates, informally expanding awareness within their professional circles. Yet, these individual relationships were felt to only go so far, with some noting challenges in changing wider perceptions and practices around more unconventional approaches in the healthcare system, such as dance.

*A pocket of people and understanding is growing*,* but there are still some healthcare professionals that really don’t see its* [dance’s] *place in terms of healthcare. *(Scottish Ballet Staff 2)

Consequently, several professionals serving in more advisory roles advocated for Scottish Ballet’s efforts to progress beyond a more “grassroots-level” [HE], decentralised approach reliant on individual advocates and focused on social prescribing. Specifically, some shared a more structured, systems-oriented strategy focused on change at the “decision-maker level” [HE] would be needed to further mainstream dance in health over the long-term, with social prescribing “on its own, not enough” [HE].

*At the moment*,* probably connecting with individuals*,* the hands-on*,* like here’s a class and that will have its own momentum and grow*,* but trying to find the right place to plug them into the system*,* should give them a strength and some security. *(Health Executive 2)

#### Maintaining cross-sector progress

Despite enthusiasm around further strategic coordination between the arts and health, challenges remained in embedding dance in healthcare at a systems level. Several professionals highlighted differences in governance and evidence standards between the health and cultural sectors as a key barrier, each operating under distinct regulatory and evaluation frameworks. Some also noted the lack of a national social prescribing framework as further limiting consistent institutional support, guidance, and pathways across health boards.

[With third-sector organisations] *there’s not the same level of clinical governance that we have at the NHS – that we can guarantee in the NHS. *(Allied Health Professional 1)

In turn, although several health sector professionals interviewed acknowledged a growing appreciation for more experiential, narrative, and qualitative methods and understandings in healthcare, certain cross-sector professionals also emphasised the need for a “business-minded” [TSE] approach, with clear, measurable impact evidenced via accepted health metrics to secure buy-in from decision-makers and funders.

*For the audiences that get to the strategic level*,* you need to be very business-minded. And I’m sorry*,* but it’s the really dirty*,* hard facts you need to think about. *(Third Sector Executive 3)

Even with “hard facts” [TSE], many professionals working across sectors felt the pressurised, “siloed” [HP] funding landscape was particularly challenging for interdisciplinary, collaborative ventures like dance on prescription. Most expressed challenges in the health sector in meeting basic care needs, limiting scope for additional investment in non-traditional interventions, which were often perceived as a “nice to have” [TSE] rather than a “need to have” [TSE] at a systems and funding level.

*It’s difficult to embrace something that is ‘new.’ So*,* ballet obviously is new*,* isn’t it? And we’ve always done certain things*,* but we haven’t done this. And just now*,* there are constraints in what we do*,* and so embracing something which is additional*,* it is not so straightforward to acquire funding. *(Health Professional 1)

In this climate, Scottish Ballet faced challenges securing service-level agreements, instead relying on individual advocates and more short-term arrangements with healthcare. As such, several professionals in more strategic roles emphasised the need for more diversified, cross-sectoral funding models to promote overall sustainability.

*It’s a really difficult financial climate to be trying to get service-level agreements or regular contracted services into the third sector* [including community and cultural sectors] *from Health and Social Care Partnerships or health boards*,* and so having to adapt our thinking to – Well*,* what might some of the funding models then look like? *(Third Sector Executive 2)

Additionally, while closer coordination with the healthcare system via social prescribing was a project priority for Scottish Ballet, some health and third-sector professionals also expressed concern around adapting to more rigid medical structures, potentially compromising the energy, flexibility, and creativity characteristic of Scottish Ballet as a cultural asset. As a result, several participants emphasised the importance of Scottish Ballet establishing a clear future vision and plan that balances connecting into healthcare pathways with preserving artistic and organisational integrity.

*I feel that Scottish Ballet is much better than the NHS about being proactive*,* about going into spaces and recognising there’s a need*,* and saying we’re going to do something about it. And I would hate for Scottish Ballet to lose that because their only mechanism is through social prescribing. You want to have control over your own direction of travel. *(Health Executive 2)

## Discussion

This study responds to recent calls for further understanding of cross-sector perspectives regarding social prescribing [18, 22, 23], not as ‘one coherent “thing” but an idea’ [13], focusing on the exploratory dance-based social prescribing pathways developed by Scottish Ballet, a national cultural asset. By drawing on the experiences and insights of professionals across the health, cultural, and third sectors and across varying levels of influence, this research highlights both the potential opportunities and challenges of coordinating cultural provisions with health and social care via a distinct, adaptable, and cross-sectoral approach to social prescribing.

Findings indicate the potential for national cultural institutions like Scottish Ballet to play a practical ‘provider’ role through their ongoing offering of dance for neurological conditions and continued contribution to more holistic, person-centred, and community-based models of care, aligning with the overarching aims and ethos of social prescribing [39]. Especially in the current UK landscape, where demand for non-clinical support is growing but third-sector resources are diminishing following COVID-19 and lacking government funding [22, 40, 41], sustained delivery of these kinds of suitable provisions is increasingly viewed as ‘fundamental’ [41] for acting as partners in social prescribing. While wider capacity and perception challenges were still noted in healthcare, Scottish Ballet’s positioning around health priorities, encouragement of first-hand engagement, and sustained cross-sector relationships helped promote understanding and buy-in from health professionals for developing dance-based prescribing pathways. Other social prescribing literature has highlighted the value of developing shared understanding [42] and ‘emotional buy-in’ [43] across partners, particularly health professionals [44]. Findings show Scottish Ballet’s strategic, direct, and sustained engagements as ways to advance understanding and buy-in around cultural provisions in health settings.

Scottish Ballet was also viewed as serving a wider role as a strategic ‘promoter’ for the arts in healthcare and social prescribing in Scotland [38, 45]. Building from their prominence in the culture sector, Scottish Ballet also internally structured their resources to externally influence the health sector, such as via strategic roles (e.g., Health Partnerships Manager), relationships (e.g., the ALLIANCE), structures (e.g., internal committees), and initiatives (e.g., Healing Arts Scotland). Most of these internal professional supports also became key external advocates, given their high emotional buy-in [43]. However, as large-scale institutions like Scottish Ballet continue deepening connections into the health sector, some suggested the need to further support the capacity of smaller-scale cultural assets, as well as clearly define role expectations and boundaries of cultural providers. These perspectives echo wider concerns about community assets assuming health and delivery responsibilities without sufficient long-term internal or external support [22, 23, 46]. In turn, findings suggest potential opportunities for cultural assets to strengthen, legitimise, and promote their provisions across health settings, while also illustrating challenges like the risks of role overextension and lacking sustainable capacity and support across culture and health sectors.

While there was general support for Scottish Ballet’s dual provider and promoter role, other challenges were noted when translating this potential into dance on prescription practice. Operationally, Scottish Ballet explored a range of social prescribing pathways, including via established primary care-based infrastructure, but the specialised neurological provisions held more limited relevance to non-specialist staff, e.g., community links workers. Further adoption was reported among specialist secondary care neurological staff backed with clinical leadership support. These differences in adoption support calls for further engagement with diverse, bespoke prescribing pathways to complement the traditional primary care-based approach [13], with potential to enhance professionals’ care of specialist patient needs via specialist community resources. Findings also reinforce evidence around the complexity of social prescribing [42, 43, 47], with any non-traditional routes needing to be sensitive to this complexity, starting with shared objectives suited to the local clinical setting and leadership. Such localised and tailored approaches may entail consideration of the most effective ways for putting arts ‘on prescription’; for example, weighing the essential yet broader remit of community links workers. As such, the effective development of arts on prescription pathways entails localised coordination between cultural and health providers to the operational needs and realities of a given professional context.

These operational-level findings also reiterate the key ‘linking’ role of health professionals [43, 48], whose engagement can be influenced by a range of factors, including perceived relevance, confidence, and patient receptiveness around provisions [42]. In the current study, positive first-hand experiences with SB Health’s provisions, including positive patient feedback and outcomes, encouraged continued adoption, complementing evidence demonstrating the significance of this patient-professional interaction in maintaining pathways [24, 42, 48]. Yet, a key rationale behind social prescribing is not only to support health outcomes, but also to improve health equity by addressing social determinants of health [39, 49]. While professional-led prescribing was felt to improve access for some individuals compared to self-referral, ongoing physical, digital, and cultural perception barriers continued to limit engagement, especially from certain more remote and socioeconomically deprived communities. Suggestions for improving access align with other evidence-based recommendations, primarily focused on further localised, responsive community-based engagement efforts by health, cultural, and third-sector providers [41]. Thereby, partners working across sectors hold important roles and responsibilities in helping ensure that social prescribing addresses health disparities alongside health outcomes [50].

On a strategic level, tensions were also identified around further connecting cultural assets like Scottish Ballet into health strategies and structures. A key challenge concerned the differing governance and evaluation standards between the arts and health sectors. For example, while those approaches and metrics most accepted in health, e.g., randomised controlled trials, are often recognised as inadequate for understanding the complex, relational nature of social prescribing [22, 43, 51], there were reportedly pressures to adhere to more conventional expectations. These differences present both a potential barrier to coordinating the arts with healthcare and a potential risk in standardisation, possibly minimising the creativity, flexibility, and nuance of arts experiences [23, 52]. Relatedly, some professionals raised concerns about embedding cultural assets too deeply in health structures, potentially compromising their creative and organisational autonomy. There were similar concerns noted in another UK study of Men’s Sheds, where closer coordination via social prescribing risked changing the natural, informal, and community-led ethos and dynamics of provisions [46]. Together, these findings indicate that drawing on traditional NHS social prescribing structures may support coordination and adoption of the arts in health; however, any developments should consider the maintenance of the artistic, organisational, and social integrity of cultural assets.

While long-term adoption in the health sector was a key aim of Scottish Ballet’s provision and promotion efforts, cross-sector perspectives reflected ongoing challenges in securing more sustainable, systems-level support. Service-level agreements have been recognised as important in the effective implementation of social prescribing [42], but such arrangements were noted as difficult to secure in the present study, given the pressurised funding landscape across health and social care in Scotland. While service-level arrangements could present one way forward, particularly with specialist services, pressurised conditions also indicate a need for further diversified, cross-sector funding models that reduce reliance on any single sector [22] and encourage more integrated, community-centred, and sustainable social prescribing [53]. Overall, as efforts to bridge cultural assets with health services progress, shared and sustained cross-sector responsibilities and investment are needed in order to support collaboration in a way that enhances each sector’s distinct role and contributions.

### Implications and recommendations

Given the small-scale, qualitative design of the present study, findings were not aimed at broad generalisability but rather at an in-depth understanding of Scottish Ballet’s approach and how this could shed light on broader cross-sectoral social prescribing models. In turn, findings could provide useful insights for other cross-sector collaborations. For cultural actors, current findings indicate that first-hand engagement opportunities for health professionals, strategically tailored to professional priorities, could help build further bridges in understanding and practice with the health sector, including for both specialist and generic arts provisions. For third-sector organisations, providing support via intermediary roles, responsibilities, and insights could help facilitate other collaborations between cultural assets and health services. For the health sector, services may benefit from expanding beyond core primary care-based social prescribing pathways, further exploring non-traditional pathways, partners, and resources to address the different, complex needs of different patient groups. Across sectors, there is also a need to consider further cross-sector, diversified funding approaches for arts on prescription, potentially expanding their reach and sustainability. Further research on these non-traditional prescribing pathways and funding models could help to illustrate other challenges, opportunities, and ways to connect the arts in health, including whether social prescribing presents the best approach for cross-sector coordination moving forward. Future research should also consider how these alternative routes affect patient access and outcomes.

### Strengths and limitations

The current study provides one of the first in-depth explorations into the role of a national cultural asset in social prescribing from the professional perspective. One strength was in terms of the diversity of the sample and viewpoints considered across sectors, geographic regions, and strategic levels. Yet, a key limitation was a sample bias towards professionals with close understanding and involvement with Scottish Ballet’s approach, especially owing to Scottish Ballet’s role in recruitment, limiting the representation from those less familiar or engaged with the project. Consequently, perspectives reflect a particular professional sample and may not represent those of wider professional groups in Scotland. Also, while Braun and Clarke acknowledge that reflexive thematic analysis can be conducted by a single researcher [35] and reflexivity was actively practiced throughout, including via memo-writing, reflection on positionality, and the iterative development of themes, analysis by a sole researcher can introduce the potential for interpretive bias. Lastly, given the study’s focus on a national cultural asset in Scotland, this has implications for the transferability of findings to smaller, local cultural providers or other contexts with different arts and health infrastructures.

## Conclusion

This study shows the potential for national cultural organisations like Scottish Ballet to act as both service providers within social prescribing pathways and wider promoters of the holistic, person-centred care ethos encouraged by social prescribing. While Scottish Ballet was viewed by professionals working across sectors as offering valuable, non-clinical provisions for those with chronic neurological conditions, challenges around professional buy-in, service-level coordination, and long-term sustainability remain evident. Findings demonstrate the need for cross-sector collaboration, understanding, and investment to support the role of cultural assets in mainstream health and social care pathways.

## Supplementary Information

Supplementary Material 1

## Data Availability

The full qualitative dataset generated and analysed during this study is not publicly available because the author considers there to be a residual risk of disclosure in the data. Please contact the corresponding author [ED] via email about obtaining access to the data.

## Abbreviations

Parkinson’s: Parkinson’s disease
MS: Multiple sclerosis
UK: United Kingdom
NHS: National Health Service
ALLIANCE: Health and Social Care Alliance Scotland
GP: General Practitioner
COREQ: Consolidated Criteria for Reporting Qualitative Research
TSE: Third Sector Executives
SBS: Scottish Ballet Staff
HE: Health Executives
HP: Health Professionals
CLP: Community Links Professionals
AHP: Allied Health Professionals
HAS: Healing Arts Scotland
WHO: World Health Organization
