# Supplementary material for: Cross-sector perspectives on the role of a UK national cultural asset in social prescribing: a qualitative study
Source: BMC Prim Care. 2026 Jan 3;27:46. doi: 10.1186/s12875-025-03156-7 (PMC12882387; doi:10.1186/s12875-025-03156-7)
Supplement: Supplementary file 1 — Supplementary Material 1 [file 12875_2025_3156_MOESM1_ESM.docx]

## Topic Guide for Interviews with Cross-sector Professionals

| Topic Domain | Sample Questions |
| --- | --- |
| Introduction | - Explanation about ethics, consent, and confidentiality - Review any participant questions - Obtain verbal permission to record |
| Background | - Can you describe your professional background and current role? - How did you come to work with Scottish Ballet? Why? - How did you become involved with the social prescribing model? - What is your role in the social prescribing model? |
| Experience | - **Practical experience** - How did the social prescribing model come about? - What does the social prescribing model look like in your practice? - How do you see SB Health integrated into the health service? - Are there any challenges to the social prescribing model? - Are there any enablers to the social prescribing model? - Can you share any insights on patient experiences/ impact? - Have there been any changes in your experience over time? - **Perceptions** - How confident do you feel in the social prescribing model? - Do you perceive any differences in SB Health’s dance offer previously versus this dance offer now ‘on prescription’? - Do any strengths come to mind in the social prescribing approach? - Do you recognise any areas for improvement in the social prescribing approach? - Have you noticed any changes in your perceptions over time? - How does your view of Scottish Ballet compare to that of others in your professional circles? - Do you have any suggestions for the future of the social prescribing model? |
| Wider Considerations | - Do you see any wider barriers to social prescribing in Scotland? - Do you recognise any wider enablers to social prescribing in Scotland? - Where do you see the role of Scottish Ballet in the social prescribing landscape in Scotland? Other arts organisations? - How can social prescribing using arts be improved looking forward? |
| Conclusion | - Is there anything else that could be relevant that you would like to share? - Thank participant for their time |
